# Extension of the viral ecology in humans using viral profile hidden Markov models

**DOI:** 10.1371/journal.pone.0190938

**Published:** 2018-01-19

**Authors:** Zurab Bzhalava, Emilie Hultin, Joakim Dillner

**Affiliations:** Dept. of Laboratory Medicine, Karolinska Institutet, Stockholm, Sweden; Oklahoma State University, UNITED STATES

## Abstract

When human samples are sequenced, many assembled contigs are “unknown”, as conventional alignments find no similarity to known sequences. Hidden Markov models (HMM) exploit the positions of specific nucleotides in protein-encoding codons in various microbes. The algorithm HMMER3 implements HMM using a reference set of sequences encoding viral proteins, “vFam”. We used HMMER3 analysis of “unknown” human sample-derived sequences and identified 510 contigs distantly related to viruses (Anelloviridae (n = 1), Baculoviridae (n = 34), Circoviridae (n = 35), Caulimoviridae (n = 3), Closteroviridae (n = 5), Geminiviridae (n = 21), Herpesviridae (n = 10), Iridoviridae (n = 12), Marseillevirus (n = 26), Mimiviridae (n = 80), Phycodnaviridae (n = 165), Poxviridae (n = 23), Retroviridae (n = 6) and 89 contigs related to described viruses not yet assigned to any taxonomic family). In summary, we find that analysis using the HMMER3 algorithm and the “vFam” database greatly extended the detection of viruses in biospecimens from humans.

## Introduction

Humans are densely populated by microbes, including viruses[1, 2]. The proportion of microbes that is viral and the composition of the metagenome seem to be altered in diseased individuals[3, 4]. However, it is possible that current metagenomics studies report only a fraction of the viruses that infect humans, as many novel viruses are continuously detected[5–10].

About 16% of all new cancer cases worldwide are attributable to infections[11]. In addition to the considerable proportion of cancers established to be caused by viruses, there are also epidemiological indications that additional cancer-associated viruses might exist. For example, several specific cancer forms are increased among individuals that have an impaired control of virus infections[12–18]. Similarly, there are multiple indications that viruses may be involved in the etiology of autoimmune diseases such as multiple sclerosis [19] and diabetes [5]. Strategies for improved detection of viruses are therefore a high priority.

Next Generation Sequencing (NGS) technologies can be used to obtain a comprehensive and unbiased sequencing of the DNA present in a sample, without the requirement of prior PCR or other amplification that requires prior information about sequences that may be present[20]. The complete sequencing of all microbiological sequences that may be present in a sample is termed shotgun metagenomics[21]. Virus discovery and detection is nowadays routinely performed in metagenomic samples [5, 7–9, 22–26].

Generally, identification of potentially viral sequences in metagenomic datasets relies on alignment-based taxonomic classifications where sequences are compared to known genomes through pairwise alignment of nucleotide/protein sequences, followed by calculating how many percent similarity they share. A drawback of this method is that public sequence databases are incomplete, especially for virus-related genomes, and metagenomic datasets might contain a large amount of sequences that have very distant homologues or no homologues at all in public databases. Indeed, a large part of the sequencing reads from *de novo* sequencing projects are classified as “unknown” [5]. Conventional alignment-based classification methods, such as BLAST[27] searches are suboptimal and thus there is an interest in the use of more sensitive algorithms, able to identify more distant homologs that may represent novel, yet unknown viruses. For example, recent studies using an alignment-free taxonomic classification method (feature frequency profiles) identified no less than 608 previously unknown and diverse ssDNA viruses in NGS datasets from seawater samples[9].

Algorithms that use profile hidden Markov models (HMMs) statistically model multiple sequence alignments for database searches. One of the advantages of HMM is that it uses position-specific scores for proteins, with penalties for insertion and deletion. In other words, it models evolutionary events which might insert or delete amino acids in genomes. Pairwise alignment tools such as BLAST use position-independent scoring systems, which makes it hard to search for similarities between distantly related microbes. HMM enables searching databases while applying multiple sequence alignments, rather than single query sequences. As the reference database for a profile HMM represent an entire sequence family (not only a single sequence), HMM can thus detect patterns that are common to a family of viruses but that may be difficult to detect when comparing to only one sequence. The HMM architecture was introduced to computational biology some 20 years ago[28], but has so far had a more limited use than BLAST-based alignments. The HMM algorithm HMMER3 has been used to search for RNA viral sequences in plasma[29], to study pathogenicity of Ebolavirus[30] and for subtyping of human influenza virus[31]. We wished to use HMMER3 with the viral profile database vFam to investigate if the overall viral ecology in human biospecimens of a variety of sources would be extended.

To this end, we used the HMM pipeline HMMER3 with vFam database for reanalysis of all contigs previously classified as “unknown” by a conventional alignment-based taxonomic classification method (based on NCBI BLAST) in NGS analyses of human samples[5–8, 10, 32–35].

## Materials and methods

### Samples and sequencing types

The metagenomic sequencing datasets were generated by Next Generation Sequencing (NGS) technologies applied to human biospecimens originating from several different patients groups, previously described in detail[6, 10, 32–37].

Shortly, all of these analyses were designed to investigate the presence of viral sequences or other microorganisms in human samples from individuals who developed diseases or from matched control subjects. For most samples, we extracted total nucleic acids, except for formalin-fixed paraffin-embedded (FFPE) biopsies where only DNA was extracted. The types of samples included serum[34], as well as fresh frozen biopsies, swabs and FFPE from skin lesions[10, 33] and from condylomata [8, 32]. Metadata of the samples used is provided in S1 Table. Sequences were obtained from the MiSeq, NextSeq and HiSeq (Illumina) sequencing platforms, as described by the manufacturer. Illumina 4000 was not used. When multiple human samples were included in the same sequencing run, the sequences were mapped to the originating sample using sequence indices, included in the Illumina adapters.

### Bioinformatics analysis

All projects were analyzed using a streamlined bioinformatics workflow, as described [38]. Shortly, the bioinformatics analysis started with quality checking, where sequences were trimmed according to their Phred quality scores. Quality checked reads were then screened against the human reference genome version 19, as well as bacterial, phage and vector sequences downloaded from GenBank using BWA-MEM (http://bio-bwa.sourceforge.net/bwa.shtml). Reads with >95% identity over 75% of their length to human, bacterial, phage and vector DNA were removed from further analysis. The rest of the sequences were normalized[39] to discard redundant data and reduce sampling variation and sequencing errors. The normalized dataset was then processed for assembly using the SOAPdenovo, SOAPdenovo-Trans[40], Trinity[41] and IDBA-UD (http://i.cs.hku.hk/~alse/hkubrg/projects/idba_ud/) assemblers into contiguous sequences (contigs). Crude reads were mapped to the assembled contigs. The use of several assembly algorithms and re-mapping of all singleton reads to assembled contigs is used to validate assembly results. Assembled contigs were then subjected to taxonomic classification by comparing them against GenBank nt and nr databases by blastn and blastx algorithms using paracel blast (www.paracel.com) to classify them as i) previously known sequences, ii) related to previously known sequences, or iii) unrelated to any previously known sequences. With blastn, a sequence was considered positive if it had a hit with e-value less than 1e-4. With blastx the e-value score used as cutoff was less than 1e-3. Table 1 shows number of contigs mapped to specific taxonomy groups.

**Table 1:** Number of contigs classified into different taxonomy groups by blastn and blastx.

Project ID	Bacteria	Human	Virus	Other
2011_1	3134	3515	251	4967
2011_2	36824	106648	29	6689
2014_1	1863	9957	81	2961
2014_2	4670	7845	348	14344
2014_3	1521	25100	41	1805
2014_4	2002	52057	129	3110
2014_5	3801	72491	561	14662
2014_6	1172	5591	86	862
2014_7	826	626	7	398
2014_8	2635	58986	247	19891
2015_1	0	66454	0	0
2015_2	570	47123	26	741
2015_3	3638	63553	263	21602
2015_4	2118	103779	383	28617
2014_A1	989	1227	11	382
2015_5_LH	0	206	0	0
2014_9	17975	1687633	353	5233
2014_14	0	1586	0	0
2014_15_SR	25299	1178299	136	7612
2013_1	143	4792	2	604
2013_2	183	3740	1	147
2012_D3	0	21	0	0
2014K1	5	647	6	120
2014_10	275	458	0	194
2014_11	6	1128	0	5
2014_12	154	31718	12	31
Total	109803	3535180	2973	134977

Column “Other” includes contigs that were classified as plants, invertebrates, synthetic, etc. We consider these as low quality contigs.

To search for distant homologs of already known viruses among the contigs classified as “unrelated to any previously known sequences” we used HMMER3[42] (the hmmsearch algorithm). Hmmsearch is designed to search sequence databases and identify remote homologues by implementing profile hidden markov models. As reference database, we used the database constructed by Skewes-Cox et al [43]. This database includes viral profile hidden Markov models (“vFams”) from all the virally annotated proteins in RefSeq [43]. The hmmsearch algorithm calculates E-value to order top sequences. Sequence was considered viral if one of its genes had hit with e-value less than 1e-5. If a sequence had hits with several virus families, the hit with the lowest E-value was chosen.

Our study is based on re-analysis of a series of previous studies on metagenomics sequencing, analysed with the bioinformatics pipeline that was most up-to-date at that time. The studies had the following Ethical Review Board (ERB) permissions: 2011/1026-31/4; 2012/1028/32; 53/2005; 612/2008; LU574-03; 104/2006; R13149, 2/2014; 2011-198-31M and 12/780-32. In the Swedish system, the Ethical Review Board (ERB) is appointed by government and chaired by a senior judge. The ERB has the authority to specify the demands on information and consent and the ERB decisions were carefully followed.

## Results

The hmmsearch command from HMMER3 algorithm and the vFam database were used to analyze a total of 6 428 566 contiguous sequences that were derived from a total of 944 human samples and classified as “unknown” by the NCBI blastn algorithm. As the same microorganism may be found in multiple specimens, some sequences may share high sequence similarity with each other, creating redundancy in the database. The HMM pipeline classified 224 605 of these sequences as virus-related (Table 2) although they had not been found to be virus-related using the BLAST searches. This database of newly detected virus-related sequences constituted 510 different unique, non-redundant contigs.

**Table 2:** Number of contigs, classified as virus-related by HMM, stratified by related virus family and types of samples. FFPE: Formalin-fixed paraffin-embedded tissue specimens.

Realated Family	Mouth	Cervix	Condyloma	Prostate secretions	Skin (FFPE)	Saliva	Serum	Skin (Fresh)
Anelloviridae	2	0	0	0	0	0	9	0
Baculoviridae	27	0	202	3	0	9	11	4
Caulimoviridae	1	0	0	0	0	0	10	0
Circoviridae	31	0	250	12	4	29	2	1
Closteroviridae	89	0	198	0	1	11	7	46
Geminiviridae	7	0	18	0	0	0	11	0
Herpesviridae	16	0	386	6	0	7	7	16
Iridoviridae	11005	1	190	62	2	6	109338	22191
Marseillevirus	155	2	598	7	1	15	18	26
Mimiviridae	556	0	951	10	315	18	102	102
Phycodnaviridae	1162	24	1223	59	561	266	4905	235
Poxviridae	67	0	129	0	0	5	6	7
Retroviridae	82	0	0	2	0	3	590	109
Unassigned	11591	111	871	27	37383	10215	7787	81
Total	24791	138	5016	188	38267	10584	122803	22818

We also reanalyzed these sequences with a more recent alignment-based algorithm (PSI-BLAST[27]). In contrast to the results from the HMMER3 algorithm, the PSI-BLAST did not find additional viral sequences over and above what had already been found with the blastn algorithm (not shown). The PSI-BLAST algorithm was taking an exceptionally long time for analyzing the same data. After days of running without giving any new hit we decided to stop the process.

The lengths of the novel HMMER3-identified virus-related contigs ranged from 500 bp to 100 000 bp (S1 Dataset). Thirty-six contigs were related to small circular viruses (Anelloviridae (n = 1) and Circoviridae (n = 35)). Eighty-nine contigs were related to described viruses that have not yet been assigned to any virus taxonomic family. The rest of the contigs were related to larger viruses such as Baculoviridae (n = 34), Caulimoviridae (n = 3), Closteroviridae (n = 5), Geminiviridae (n = 21), Herpesviridae (n = 10), Iridoviridae (n = 12), Marseillevirus (n = 26), Mimiviridae (n = 80), Phycodnaviridae (n = 165), Retroviridae (n = 6) and Poxviridae (n = 23).

**Fig 1:**
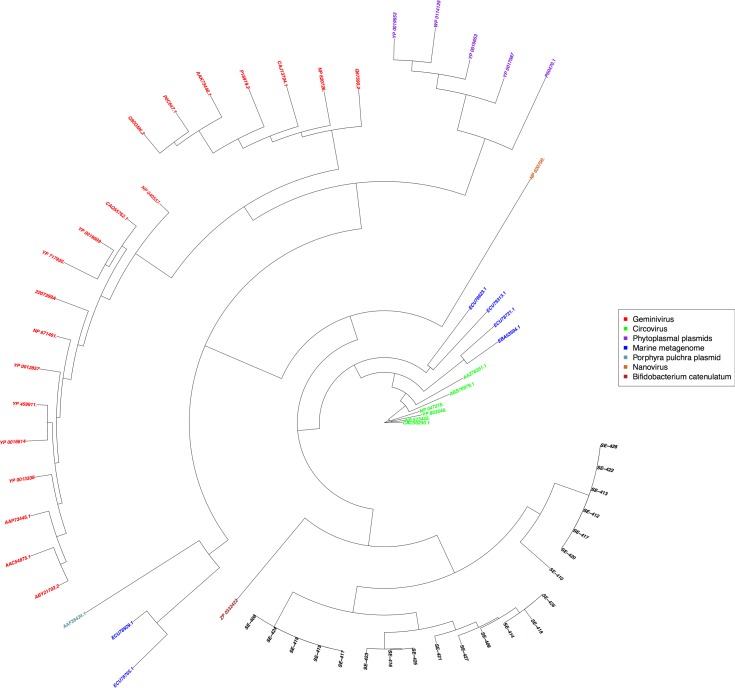
Maximum likelihood phylogenetic tree (PhyML v3.0 www.atgc-montpellier.fr/phyml/) based on the RCR Rep proteins from genbank and 21 previously not described Rep proteins related to *Circoviridae*, that were found in the present study (shown in black color with the prefix SE).

To investigate if the classification of the new contigs would be the same using other methods, a phylogenetic tree (Fig 1) was constructed based on the 21 rolling circle replication (RCR) Rep proteins identified in this study and the already known RCR Rep proteins from the PFAM database. The tree depicts segmentation of Rep proteins into several distinct groups. The newly identified RCR Rep sequences form a distinct subgroup (that also contains a sequence from Bifidobacterium catenulatum) that is quite distant from all other subgroups except from the known Circoviridae, which are in the center of the tree.

We also analyzed sequences previously classified as virus-related by the BLAST-based taxonomic classification pipeline, in order to determine the ability of HMM to identify also closely related viruses. Out of 6482 viral contigs identified by the BLAST-based pipeline, the viral HMM pipeline identified 2041 of them as viral. The average nucleotide length of these double-detected viral contigs was >1000bp. The viral contigs identified by the BLAST-based pipeline, but missed by HMM had <500 nucleotides average length. The average length of the contigs that were classified as unknown by the BLAST-based pipeline but re-classified as viruses by HMM had a much higher average length (mean = 3362, min = 501 max = 106 392) than that of the contigs that still remained “unknown” also after analysis with the HMM pipeline (mean = 365, min = 200, max = 14562).

To estimate specificity, we ran the pipeline on sequences that were labeled as “bacterial” or “human” by NCBI Blast. Altogether, 0.1% and 1% of these sequences, respectively, were instead classified as virus-related by viral HMM.

All sequences that were previously classified as unknown by NCBI BLAST and re-classified as viruses by the pipeline were compared to the pfam database[44] to search for proteins conserved in viruses. Contigs that were classified as distant homologs to big viral families commonly contained i) genes that encoded leucine rich repeats, which are present in approximately 20 000 proteins both from viruses and eukaryotes[45] ii) methyltransferase motifs that are characteristics for Chlorella viruses[46] iii) tetratricopeptide and Ankyrin repeat motifs that are typical for mimivirus and EsV-1 genomes[47, 48]. Contigs related to small circular viruses contained genes that encoded the viral hallmark genes of these viruses[48] (Table 3). For example, among the contigs related to “Circoviridae”, 21/35 contained a sequence similarity to the putative Rolling-circle replication initiation endonuclease, a characteristic of small ssDNA viral families. Six of them also contained SpoIIIE/FtsK motifs. This protein is essential for viral DNA packaging and conjugation[49]. Finally, several sequences typical for helicases were detected. Helicases are essential for viral genome replication[50].

**Table 3:** Number of different contigs detected, by the most related virus families identified using HMM and by existence of typical protein sequence motifs.

	Tetratricopeptide repeat	Leucine Rich Repeat	Helix-turn-helix	Ankyrin repeat	Methyltransferase domain	Helicases	Rep-like domain	FtsK/SpoIIIE family	Reverse transcriptase	Satellite tobacco necrosis virus coat protein
Baculoviridae	0	0	0	0	0	9	0	0	0	0
Caulimoviridae	0	0	0	0	0	0	0	0	0	0
Circoviridae	0	0	7	0	0	0	21	6	1	0
Closteroviridae	0	0	9	0	0	1	0	0	0	0
Geminiviridae	0	0	0	0	0	0	1	0	0	9
Herpesviridae	0	0	2	0	0	0	0	0	0	0
Iridoviridae	15	0	0	0	0	6	0	0	3	0
Malacoherpesviridae	0	0	0	0	0	0	0	0	0	0
Marseillevirus	9	0	5	0	0	0	0	0	0	0
Mimiviridae	0	53	20	27	10	6	0	1	0	0
Phycodnaviridae	15	0	38	10	16	18	0	1	0	0
Poxviridae	0	43	1	0	3	2	0	0	0	0
Retroviridae	0	0	0	0	0	0	0	0	2	0
Unassigned	0	0	5	25	4	3	0	0	0	21

Previously described viruses that have not yet been assigned to any recognized virus genera, but have sufficient characteristics to be distinguished from existing ones are referred to as “unclassified viruses” (http://ictvdb.bio-mirror.cn/Ictv/fsunass.htm). Twenty-one of the contigs classified as belonging to this group contained sequences coding for a protein distantly homologous to the Satellite tobacco necrosis virus coat protein.

### Benchmarking of the viral HMM pipeline

In order to evaluate the performance of the viral HMM pipeline, we used simNGS and simLibrary (http://www.ebi.ac.uk/goldman-srv/simNGS/#about) tools (the default settings of these tools were used) to simulate sequencing reads from 100 000 different genomes from Genbank including bacteria (n = 15 000), plants (n = 5 000), humans (n = 70 000) and viruses (n = 9 646). De-novo assembly resulted in a total of 55 274 contigs, from which human contigs were subtracted using BLAST in a similar manner as for the main study. The experiments were performed four times with similar results. A database with 185 contigs known to be assembled from bacteria, 4 879 contigs originating from plants and 10 789 originating from viruses was obtained.

The database with contigs of known origin was subjected to HMMER3 analysis in the same manner as in the main study. The viral HMM pipeline classified 8 113 contigs as viral. Among these, 96% were truly of viral origin and the rest (4%) were false positives, mostly originating from non-human cellular organisms. The pipeline had 100% accuracy when identifying ssDNA viruses such as Circoviridae, Anelloviridae, Parvoviridae, as well as some dsDNA viruses such as Papillomaviridae and Polyomaviridae. However, the proportion of true positives dropped to 3% in case of Mimiviridae. In most cases, the viral HMM pipeline confused this viral family with plant genomes. Although none of the samples in this study came from plants, it can not be excluded that some of the Mimiviruses found in this study came from plants, as plants may occasionally contaminate human samples.

## Discussion

As e.g. shown in Table 2, we find that the viral ecology in human biospecimens is much more diverse than what is detected using conventional alignment-based bioinformatics algorithms such as BLAST or PSI-BLAST. This finding was obtained using an already existing bioinformatics algorithm (HMMER3, based on profile Hidden Markov Models). More than 500 different previously unknown potentially viral sequences, missed by conventional BLAST-based analysis, are reported here. Most of these sequences were related to large viruses such as Mimiviridae and Herpesviridae, but many relatives of small circular viruses such as Circoviridae, were also identified.

We find that profile HMM is particularly powerful when the contig is relatively long, mostly detecting homologs that had a length >1000bp. Only one third of viral contigs detected using BLAST-based algorithms were re-detected using HMM. This suggests that HMM-based analysis should not replace BLAST-based analyses, but should be regarded as a complement for detecting distantly related similarities among longer contigs.

Some ssDNA viruses detected by us were closely related to sequences identified from seawater samples, using another alignment-free taxonomic classification method[9]. The natural history of these newly discovered “environmental” viruses is not known and most of them are not yet assigned to genera or higher taxa. Many microbes infecting humans, including viruses, can find their way into sewage systems and eventually be shed into the sea[51, 52], leaving the possibility open that some of the “environmental” viruses may be of human origin.

Among the dsDNA viruses detected, we found several that belong to families already known to infect humans such as Mimiviridae and Poxviridae.

The vFam database, used as reference in the current study, includes all known viral proteins in RefSeq. As the database grows with novel viruses discovered, the profile HMM method will become even more effective to detect yet unknown potentially viral sequences. As viruses are underrepresented in current genomic reference databases, accurate and realistic estimation of the proportion of viral DNA in metagenomics is a challenge. Thus, further development of viral sequence classification and abundance estimations methods is essential.

There are also other tools and protocols available for detecting viral genomes in microbial genomic data. The tool Virsorter[53] uses a reference set consisting of viral proteins from the RefSeq database (similar to pVOGS[54]) but this tool and its database are mainly designed to detect viruses that infect microbes (archaea and bacteria).

In a published protocol for virus sequence discovery for metagenomic data, Paez-Espino et al used a viral hmm database including thousands of viral proteins for detecting viral sequences and grouping them into viral clusters[55]. However, this pipeline does not allow annotation of identified viral sequences at species (family) level. One of the advantages of the vFam database is that it includes annotation files, which makes the annotation process easier, faster and enables easy investigation of the viral species that are present in human samples.

Evaluation of the HMMER3 pipeline with vFam database with simulated sequencing reads showed that it has very high accuracy for identification of viral contigs. Simulated data analyzed with the algorithm included bacterial, plant, and viral genome. The proportion of correctly classified viral families was >99% for ssDNA and most dsDNA viral families. However, performance for identifying a few viral families was worse, particularly for Mimiviridae, probably due to the fact that the Mimivirus genome encodes some genes previously considered as exclusive to cellular organisms[56] and greatly resembling them. Thus, even though the algorithm has very high accuracy, unexpected presence of plants in human biospecimens may result in ambiguous results. For samples of human origin, human sequences will of course be subtracted. We suggest that bioinformatics pipelines seeking to characterize the viral ecology in human biospecimens should also include subtraction of all known non-viral genomes that could conceivably contaminate the samples, for example from food, pets or parasites.

The possibility of contamination should always be considered in a sequencing study. As we used multiple contamination controls (water and blank paraffin blocks), contamination in the laboratory was probably not a major problem. However, specimens may also be biologically contaminated already before processing. Several sequences found were related to viruses not known to infect humans (e.g. Iridoviridae, Baculoviridae and Closteroviridae) but it should be emphasized that the new sequences detected were merely sequences whose closest related sequence were among these virus families, which does not mean that the new sequences necessarily belong to these families. Thus, possible explanations for the presence of these new sequences related to non-human viruses include i) new virus with unknown biological behavior ii) “biological contamination” of the specimens and iii) incomplete HMM reference database where the closest relative to these sequences was not present.

The 5 contigs related to Closteroviridae are difficult to explain, as Closteroviridae are only known to infect plants and are also ssRNA viruses. Although we extracted total nucleic acids for the samples where these were detected, we did not use reverse transcription to cDNA. The similarity was quite strong (e-values up to 10^−60^) so that these new sequences are indeed related to Closteroviridae seems likely.

In conclusion, the viral ecology in human biospecimens appears to be much more diverse than previously appreciated. This diverse ecology was readily revealed using an existing bioinformatics pipeline (HMMER3 with vFam database) applied to sequences obtained from a standard NGS analysis using the Illumina platform. Further studies of these putative viruses should therefore be straightforward and may be important for continued elucidation on the role of viruses in health and disease of humans.

## Supporting information

S1 Dataset Nucleotide sequences of 510 potentially novel viral sequences. (CSV)

S1 Table Metadata of the samples. (XLSX)
